# Carbon hybridized halloysite nanotubes for high-performance hydrogen storage capacities

**DOI:** 10.1038/srep12429

**Published:** 2015-07-23

**Authors:** Jiao Jin, Liangjie Fu, Huaming Yang, Jing Ouyang

**Affiliations:** 1 Centre for Mineral Materials, School of Minerals Processing and Bioengineering, Central South University, Changsha 410083, China

## Abstract

Hybrid nanotubes of carbon and halloysite nanotubes (HNTs) with different carbon:HNTs ratio were hydrothermally synthesized from natural halloysite and sucrose. The samples display uniformly cylindrical hollow tubular structure with different morphologies. These hybrid nanotubes were concluded to be promising medium for physisorption-based hydrogen storage. The hydrogen adsorption capacity of pristine HNTs was 0.35% at 2.65 MPa and 298 K, while that of carbon coated HNTs with the pre-set carbon:HNTs ratio of 3:1 (3C-HNTs) was 0.48% under the same condition. This carbon coated method could offer a new pattern for increasing the hydrogen adsorption capacity. It was also possible to enhance the hydrogen adsorption capacity through the spillover mechanism by incorporating palladium (Pd) in the samples of HNTs (Pd-HNTs) and 3C-HNTs (Pd-3C-HNTs and 3C-Pd-HNTs are the samples with different location of Pd nanoparticles). The hydrogen adsorption capacity of the Pd-HNTs was 0.50% at 2.65 MPa and 298 K, while those of Pd-3C-HNTs and 3C-Pd-HNTs were 0.58% and 0.63%, respectively. In particular, for this spillover mechanism of Pd-carbon-HNTs ternary system, the bidirectional transmission of atomic and molecular hydrogen (3C-Pd-HNTs) was concluded to be more effective than the unidirectional transmission (Pd-3C-HNTs) in this work for the first time.

Environmental concerns regarding the use of fossil fuels and their predicted exhaustion are globally important issues. Hydrogen has attracted attentions as one of the most promising substitute for the fossil fuels for the advantages of sustainable, renewable energy, and abundant energy supply with the additional benefit of potentially allowing the production of zero emission vehicles. However, the possible utilization of hydrogen faces the problems of production, transportation, and storage^1^. In particular, developing a safe and efficient hydrogen storage system at room temperature and atmospheric pressure is much more challenging. Having recourse to gas compression, cryogenic liquids, and hydrogen storage materials, such as metal hydride, chemical hydride, metal-organic frameworks, and adsorbed materials are the general technologies for hydrogen storage^2^,^3^,^4^. However, gas compression and cryogenic liquids face the problems of high cost, low energy efficiency, and high requirements for the equipments. Most of materials for hydrogen storage suffer from these drawbacks of poor adsorption/desorption reversibility, inherent slow kinetics, thermodynamic energy inefficiency, secondary pollution caused by the reaction products and high costs of production and regeneration^4^. In recent years, sorbent approaches of hydrogen storage have all attracted considerable attentions. Hydrogen storage materials with high gravimetric and volumetric densities must be developed to achieve economical feasibility. Ao and Peeters applied density-functional theory and study hydrogen adsorption on graphene with Al atoms, whose hydrogen storage capacity was extremely high. They also determined that the favorite adsorption configuration of Al atoms on single side and on both sides of a graphene layer^5^. Hydrogen storage by adsorption represents one potential strategy for effective and relatively safe hydrogen storage^6^,^7^. Physisorption on solid substrates is attractive mainly because of its flexibility in enabling subsequent release of the adsorbed gas, if required^8^. In particular, porous minerals are attractive choices because of their low cost, long-life cycle, relative high surface area, high durability, and hydrogen adsorption/desorption ability at room temperature when compared with other hydrogen storage materials^9^,^10^,^11^. Nanotubes have been investigated extensively for potential application based on their unique dimensions and structure^12^,^13^.

Natural halloysite, Al_2_Si_2_O_5_(OH)_4_·2H_2_O, a hydrated layered aluminosilicate of the kaolinite group, consists of hollow cylinders, formed by multiple rolled layers, with submicrometer dimensions. Natural halloysite nanotubes (HNTs) feature exceptional physical and chemical properties, such as relatively high specific surface area, high porosity, and high cation-exchange capacity. HNTs is one of the most perfect candidates for hydrogen storage in view of the high porosity, large surface area, strong adsorbability, and excellent characteristics of the natural mineral^13^,^14^,^15^. However, these adsorbed materials are not sufficiently effective to represent the final solution of hydrogen storage^16^. The basic idea is to find appropriate metals to modify the adsorbed materials aiming at enhancing hydrogen adsorption proceeds via spillover mechanism^17^. Spillover has been defined as the transport of an active species adsorb on one site to another site that would not typically adsorb the active species at the prevailing conditions. Wang *et al.* analyzed the hydrogen isotherms and isosteric heat of adsorption of Ni-B nanoalloy-doped three-dimensional graphene material, suggesting that doping with an appropriate amount of Ni-B nanoalloys could result in the dissociative chemisorption of hydrogen molecules by spillover to achieve the high hydrogen storage capacity^18^. Hydrogen storage by spillover is a promising approach to enhance the hydrogen storage capacities in nanostructured materials including carbon nanomaterials, zeolite, mineral materials, and metal-organic framework^10^,^11^,^17^,^19^,^20^,^21^. Meantime, carbon or carbon hybrid materials are of very high technological importance because of their mechanical, electronic, as well as molecular adsorption and sieving properties^22^. Many kinds of carbon materials, such as activated carbons, carbon nanofibres and carbon nanotubes have been concluded as potential hydrogen storage materials during these decades^23^,^24^,^25^,^26^,^27^. Graphene with its unique structural, electronic, thermal, and mechanical properties has been identified as a promosing candidate for hydrogen storage, especially doped graphene^5^,^28^. In addition to their high adsorption capacity and high adsorption/desorption rates, the carbon materials have to be predominantly microporous and have to provide an adequate surface chemistry and good mass transfer properties. Incorporation of carbon into another matrix material to form a new binary support material for hydrogen spillover can be more effective for hydrogen storage^19^,^20^.

The present study aimed at investigating an appropriate method to synthesize novel hybrid nanotubes of carbon and HNTs, and determine if these hybrid nanotubes produced any beneficial effects on hydrogen adsorption capacities. Different carbon:halloysite (C:HNTs) mass ratios were selected to investigate the influence of occurrence states of carbon in HNTs on the morphology and hydrogen adsorption capacities at room temperature were examined. Palladium was selected as the noble metal for the promotion of hydrogen adsorption capacities. Moreover, different kinds of spillover mechanisms in the hybrid nanotubes were examined. The hybrid nanotubes were indicated to be promising physisorption-based materials for hydrogen storage at room temperature.

## Results

**Figure 1:**
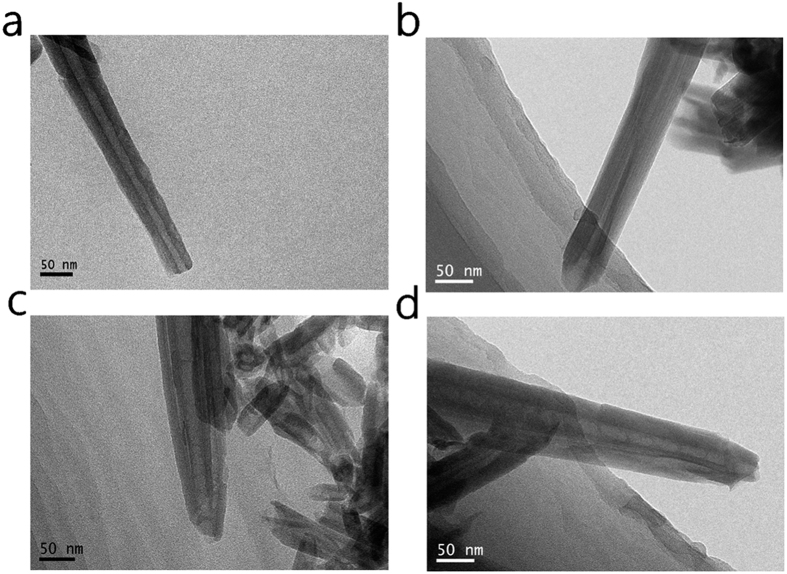
Morphologies of the samples. TEM images of (**a**) HNTs, (**b**) 1C-HNTs, (**c**) 3C-HNTs and (**d**) 5C-HNTs.

TEM images with different magnifications of HNTs and *x*C-HNTs (*x* = 1, 3 and 5) are shown in Fig. 1 and Figure S1. The HNTs sample features cylindrical hollow tubes averaging of 0.7 ~ 2 μm in length, with an external diameter of 30 ~ 75 nm, and an internal diameter of 10 ~ 30 nm (Fig. 1a). After incorporation with carbon, the characteristic tube morphology of the original HNTs remained, but both the external diameter and the internal diameter have changed. For 1C-HNTs, the size of external diameter increases, while that of the internal diameter decreases when compared with original HNTs (Fig. 1a ~ b and Figure S1b, c). The size of external diameter increases more. There is no obvious carbon layer for 1C-HNTs, but a carbon layer with the thickness of 5 ~ 10 nm can be clearly identified for 3C-HNTs sample sample (Fig. 1c), and nanotubes of 3C-HNTs become much more thicker (Figure S1d). As the content of carbon increases, the carbon layer becomes much thicker for 5C-HNTs, and there are plenty of carbon sections in the internal of tube (Fig. 1d). The incorporations of carbon in HNTs mostly form carbon layer outside the external surface of HNTs. A portion of carbon imports into the inner hollow of HNTs and form sections of carbon for 5C-HNTs.

**Figure 2:**
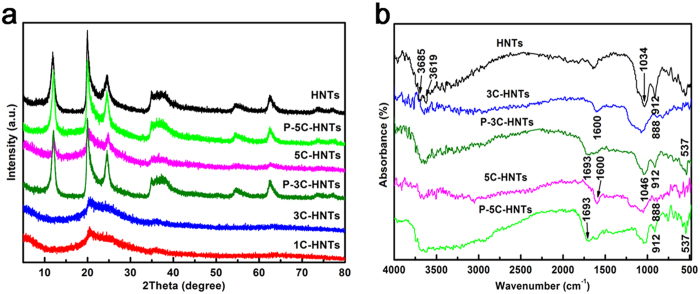
Crystallization and spectra of the samples. (**a**) XRD patterns of HNTs, *x*C-HNTs together with the precursors of 3C-HNTs and 5C-HNTs and (**b**) FTIR spectra of 3C-HNTs and 5C-HNTs together with the precursors compared with HNTs.

The XRD patterns of HNTs, *x*C-HNTs together with the precursors of 3C-HNTs and 5C-HNTs (named as P-3C-HNTs and P-5C-HNTs) are shown in Fig. 2a. The hydrothermal reaction does not affect the structure of HNTs as the precursors feature the comparable XRD patterns as HNTs. In contrast, the characteristic diffraction peaks of halloysite are absent in the 1C-HNTs and 3C-HNTs samples, and the broad peak centered at 2*θ* = 20 ~ 25° indicates that the presence of silica and carbon in the 1C-HNTs and 3C-HNTs samples are amorphous caused by calcinations at 600 °C. As the content of carbon increase, the three-strongest characteristic diffraction peaks of halloysite maintain for the 5C-HNTs sample. This may be due to the thickest carbon layer protect the structure of HNTs from changing by the thermal treatment. FTIR spectra were used to investigate the differences of structure and surface characteristics between the hybrid nanotubes and the precursors compared with pristine HNTs (Fig. 2b). The characteristic bands at 3685 cm^−1^ and 3619 cm^−1^ are due to the Al-OH stretching vibration, which are clearly seen in the HNTs sample. But the Al-OH stretching bands at 3685 cm^−1^ and 3619 cm^−1^ disappear after the incorporation of carbon for 3C-HNTs and 5C-HNTs, indicating the destroy of the gibbsite octahedral sheet of halloysite both in the hybrid nanotubes. The characteristic bands at ~3650 cm^−1^ in the cases of P-3C-HNTs and P-5C-HNTs could be due to the absorbed water from hydrothermal synthesis method. At the same time, the bands at 888 cm^−1^ for P-3C-HNTs and P-5C-HNTs, and 537 cm^−1^ for 3C-HNTs and 5C-HNTs appear from the carbon backbone framework. The peaks at 1693 cm^−1^ for P-3C-HNTs and P-5C-HNTs samples correspond to the -COOH indicating the formation of polysaccharide layer for carbon. The polysaccharide layer for carbon is due to the incomplete carbonization for sucrose during the hydrothermal procedure. There are new peaks at 1600 cm^−1^ for 3C-HNTs and 5C-HNTs samples represent the conjugated double bonds as the complete carbonization of sucrose after 600 °C thermal treatment under nitrogen atmosphere. The peak at 1034 cm^−1^ corresponds to the Si-O net work (Si-O-Si and O-Si-O) for HNTs. The broad band at 1034 cm^−1^ shifts to 1046 cm^−1^ due to the Al was covered with carbon after the incorporation of carbon. The band at 912 cm^−1^ represents Al-OH bending vibration which is clearly seen in the HNTs, P-3C-HNTs and P-5C-HNTs samples. But the Al-OH bending vibration disappears in the 3C-HNTs and 5C-HNTs samples due to the thermal treatment. The broad band between 1644 cm^−1^ to 3600 cm^−1^ is attributed to the absorbed water. The peak around 700 cm^−1^ corresponds to Al-OH translational vibration. The adsorbed bands are more obvious for the precursors because of aqueous-phase synthesis for the hydrothermal procedure, while the adsorbed bands of 3C-HNTs and 5C-HNTs samples decrease because of the thermal treatment.

Nitrogen adsorption-desorption isotherms of HNTs and *x*C-HNTs, together with corresponding BJH pore size distributions are shown in Figure S2. All the isotherms exhibit type II with distinct type *H3* hysteresis loop in the relative pressure (*p/p*_*0*_) range of 0.7–1.0 together with a *H2* hysteresis loop in the relative pressure range of 0.4–0.7^29^. According to the standard setup by International Union of Pure and Applied Chemistry (IUPAC)^30^, hysteresis loop is the representative of capillary condensation occurred among the ordered porous channels or interspaces of particles. The porous performance parameters are listed in Table 1. Owing to the thermal treatment and incorporation of carbon, the hybrid nanotubes have some structural shrinkage which leads to the decrease of BET surface areas compared to HNTs.

**Figure 3:**
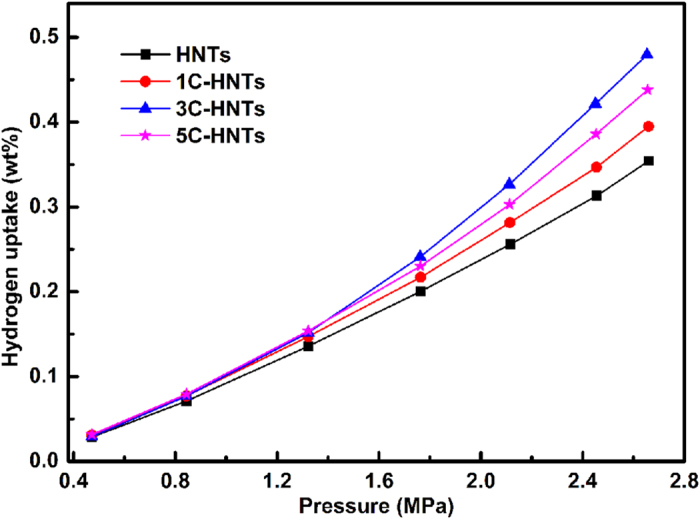
Hydrogen adsorption capacity of the samples. The change of pressure of hydrogen with the reaction proceeding.

Figure 3 shows the hydrogen adsorption isotherms of the HNTs and hybrid nanotubes samples measured at 25 °C. All the obtained hydrogen adsorption isotherms featured a typical physical adsorption profile: hydrogen adsorption values linearly increase with an increase in pressure. This indicates that hydrogen adsorption on the HNTs and *x*C-HNTs samples proceeds via a physisorption process. The hydrogen adsorption capacities of HNTs and *x*C-HNTs samples are among the highest reported values, as achieved by known sorbents. For instance, HNTs have a hydrogen adsorption capacity of 0.35 wt%, measured at room temperature and 2.65 MPa. Halloysite nanotubes have natural aluminosilicate characteristic with large surface area, high porosity, tunable surface chemistry, and plenty of hydroxyl, which could be considered as one of the best candidates for hydrogen storage^29^,^31^. The interlayer spacing is a little larger than the molecular kinetic diameter of hydrogen molecular^31^, so it allows the hydrogen molecules to enter into the interlayer which leads to a considerable hydrogen adsorption capacity. Moreover, the special nano-scale hollow cylinders could provide excellent channels to avoid hydrogen blocking compared with other minerals^9^. The hydrogen adsorption capacities of 1C-HNTs, 3C-HNTs and 5C-HNTs are 0.39 wt%, 0.48 wt% and 0.43 wt%, respectively. The uniform channels of the HNTs and *x*C-HNT samples may be more favorable for hydrogen transport and storage in contrast to those obtained from synthetic silica nanotubes under the same pressure^32^. Hybrid nanotubes got significantly improvement of hydrogen adsorption compared with natural HNTs. It can be deduced that large surface areas are beneficial towards promoting hydrogen uptake which proceeds through a physisorption mechanism. Surface area is not the predominant factor in determining the hydrogen adsorption capacity of the HNTs and the *x*C-HNT samples. Although high surface areas are important for hydrogen storage by adsorption on solids, it would appear that it is essential that not only the physical, but also the chemical properties of the adsorbents have to be considered in the quest for the hybrid nanotubes, with high hydrogen storage capacities^33^. The chemical properties of the adsorbents, large surface area, plenty of mesopores, and appropriate small average pore size are in favor of hydrogen adsorption^11^.

**Figure 4:**
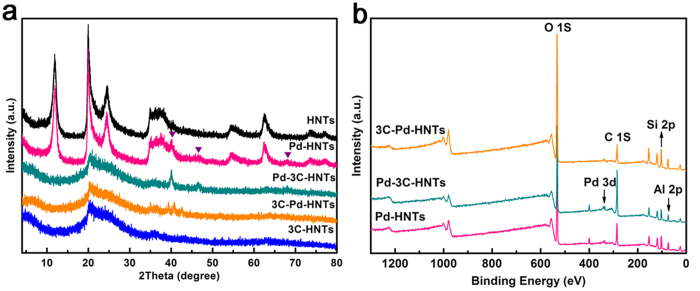
Crystallization and spectra of the samples. (**a**) XRD patterns of HNTs, Pd-HNTs, 3C-HNTs, Pd-3C-HNTs, and 3C-Pd-HNTs, and (**b**) XPS spectra of Pd-HNTs, Pd-3C-HNTs and 3C-Pd-HNTs.

Transition metals (Pd, Pt, Ni, etc.) can be doped to enhance the storage capacity via a hydrogen spillover mechanism. Palladium (Pd) is an archetypical hydrogen storage metal. It is possible to enhance hydrogen adsorption capacity through chemisorptions or spillover mechanism for the sample loaded with Pd. So Pd was selected as the noble metal for the promotion of hydrogen adsorption capacities during this part. The characteristic diffraction peaks of halloysite (JCPDS card NO. 29–1487) exist in the HNTs and Pd-HNTs samples. The Pd-3C-HNTs and 3C-Pd-HNTs samples feature the same broad peak of amorphous silica and carbon centered at 2*θ* = 20 ~ 25° (Fig. 4a). In addition, three new reflections centered at 2θ = 40.1°, 46.6°, and 68.1° were observed for Pd-HNTs, Pd-3C-HNTs and 3C-Pd-HNTs samples; these correspond to the (111), (200), and (220) lattice planes of Pd, respectively (JCPDS NO. 65-2867). But the intensity of these peaks reduces and there are some shifts for the 3C-Pd-HNTs sample, which probably due to the carbon layer coated around the Pd nanoparticles. Further evidence for the purity and composition of the product was obtained by XPS spectra. The wide-scan XPS specta of Pd-HNTs, Pd-3C-HNTs and 3C-Pd-HNTs (Fig. 4b) show indexed peaks corresponded to Si, O, Al and C elements. The high-resolution XPS spectra of Pd 3d region are shown in Figure S3. The Pd 3d_5/2_ and 3d_3/2_ peaks located around 335.12 eV and 340.37 eV belong to zero-valent palladium in Pd-HNTs. The Pd 3d_5/2_ and Pd 3d_3/2_ electronic states of Pd(0) shift little to higher binding energy for Pd-3C-HNTs and 3C-Pd-HNTs, which indicates the imcomplete reduction caused by more complex process.

Nitrogen adsorption-desorption isotherms of HNTs, Pd-HNTs, 3C-HNTs, Pd-3C-HNTs and 3C-Pd-HNTs together with corresponding BJH pore size distributions are shown in Figure S4. All the isotherms exhibit the same obvious type *H3* hysteresis loop in the relative pressure (*p/p*_*0*_) range of 0.7–1.0 together with a *H2* hysteresis loop in the relative pressure range of 0.4–0.7 with HNTs and 3C-HNTs substrates, which indicating the Pd incorporation does not affect the porous structure of substrates. The type *H3* hysteresis loop, which does not have any adsorption plateau at high relative pressure, is an evidence of macropores and interspaces of different tubes in all the samples. The middle section of the isotherms in the relative pressure range of 0.4–0.7, linkable to capillary condensation, is caused by mesoporous adsorption, which indicates the existing of textural mesopores in the samples. The pore size distribution of Pd-HNTs features the similar characteristic with HNTs. And the pore size distributions of Pd-3C-HNTs and 3C-Pd-HNTs feature the same tendency with 3C-HNTs. For the samples of Pd-HNTs and Pd-3C-HNTs, which are directly loaded with Pd nanoparticles on HNTs and 3C-HNTs, both the BET surface areas and pore volumes decrease (Table 1), while the average pore sizes increase compared with their original substrates, but the pore volume and average pore size increase for the 3C-Pd-HNTs sample, for which the Pd nanoparticles are between the carbon layer and HNTs. Meanwhile, the 3C-Pd-HNTs sample has the advantage of high BET surface area, which can compare favourably with HNTs. This is probably due to the Pd nanoparticle between the carbon layer and HNTs, which can play the role of supporting the carbon layer. Interspaces would generate as the carbon layer was separated from HNTs by Pd nanoparticles.

**Figure 5:**
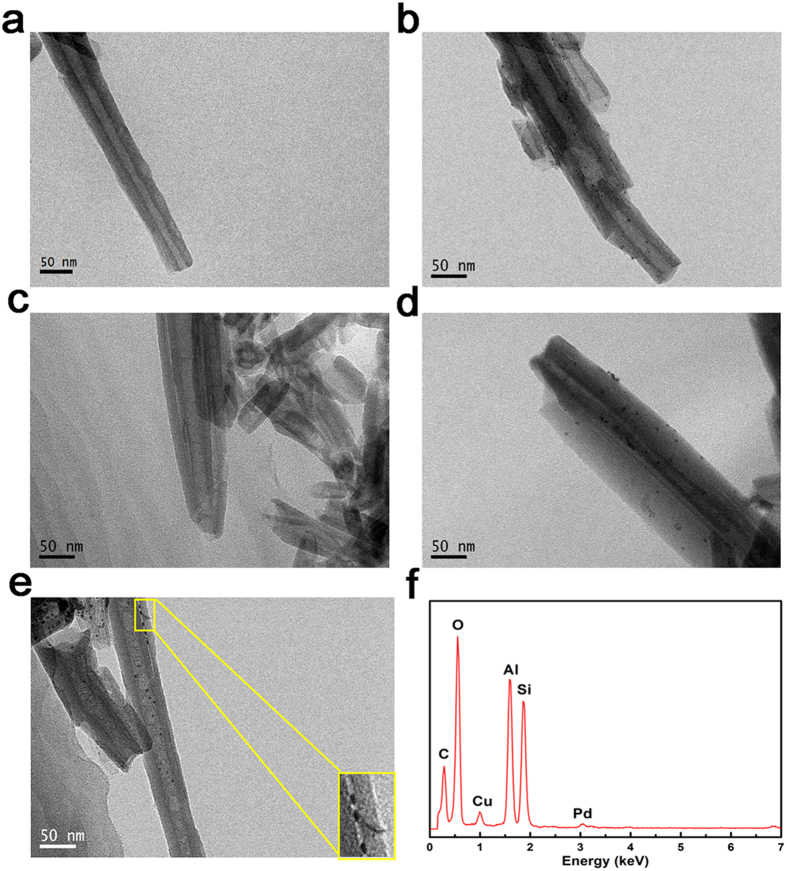
Morphologies of the samples. TEM images of (**a**) HNTs, (**b**) Pd-HNTs, (**c**) 3C-HNTs, (**d**) Pd-3C-HNTs, (**e**) 3C-Pd-HNTs (insets of e is the partial enlarged detail of 3C-Pd-HNTs) together with (**f**) the EDX spectra of 3C-Pd-HNTs.

Halloysite comprises naturally occurring aluminosilicate nanotubes with a 1:1 Al:Si ratio, which are mainly composed of O, Si, and Al elements. Black spots in the samples of Pd-HNTs, Pd-3C-HNTs and 3C-Pd-HNTs are confirming to be Pd nanoparticles through the EDX spectrum (Fig. 5f). After assembly with Pd nanoparticles, the characteristic hollow tube morphology of the original HNTs has been retained, and Pd nanoparticles with several nanometers in diameter are highly dispersed both on the external surfaces and in the internal tubes (Fig. 5b). Pd nanoparticles with several nanometers in diameter are highly dispersed both on the middle section and the outer edge for Pd-3C-HNTs sample (Fig. 5d). Combined the TEM results with the synthetic process, Pd nanoparticles are supposed to highly disperse on the outer carbon layer. For the 3C-Pd-HNTs sample, Pd nanoparticles are highly dispersed just in the middle section of the tubular structure (Fig. 5e). And there is an obvious boundary line between the carbon layer and HNTs. Pd nanoparticles are dispersed between the carbon layer and HNTs, which clearly displays in the inset of Fig. 5e. The interlayer spacing of HNTs is a little larger than the molecular kinetic diameter of hydrogen molecular^29^,^31^, which can ensure the Pd particles between carbon layer and HNTs to contact with hydrogen gas. Moreover, the special nano-scale hollow cylinders of HNTs could provide excellent channels to avoid hydrogen blocking^9^. The significant difference between Pd-3C-HNTs and 3C-Pd-HNTs is the location of Pd nanoparticles. Pd nanoparticles were outside the carbon layer for Pd-3C-HNTs, while Pd nanoparticles were between the carbon layer and HNTs for the sample of 3C-Pd-HNTs.

## Discussion

**Figure 6:**
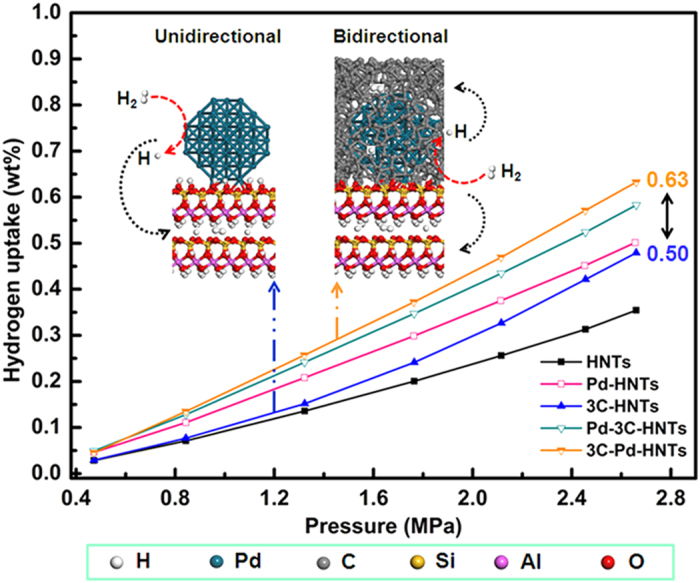
Hydrogen adsorption capacity of the samples. Hydrogen adsorption isotherms of different samples and different spillover of hydrogen from Pd metal to the carbon and HNTs supports.

The hydrogen adsorption isotherms of different samples measured at 25 °C are shown in Fig. 6. All the obtained hydrogen adsorption isotherms featured the typical physical adsorption profile, indicating that hydrogen adsorption on the samples mainly proceeds via the physisorption process. Large surface areas are beneficial to promoting hydrogen uptake that proceeds through a physisorption mechanism^34^. Clearly, all the Pd-doped samples show enhanced storage capacity compared to their suitably plain materials. The enhancement of hydrogen storage should be attributed to the spillover of hydrogen from Pd nanoparticles to the receptors, but not to the surface area difference since the Pd-doped materials have lower surface areas than the plain materials. Despite the low surface area of the Pd-HNTs sample, Pd-HNTs showed an enhanced hydrogen adsorption capacity (i.e., 0.50 wt% at room temperature and 2.65 MPa), which could be due to the presence of the Pd nanoparticles in the sample that are capable of efficiently promoting the hydrogen adsorption capacities. The enhanced hydrogen adsorption proceeds via a chemisorption or spillover mechanism^35^,^36^. Surface area is not the predominant factor in determining the hydrogen adsorption capacity through the chemisorption or spillover mechanism^35^,^37^. Doping with transition metals (e.g., Pd, Pt, Ni) is an effective way to enhance the hydrogen storage capacity, as demonstrated by several literatures^38^,^39^. Transition metals have strong ability in storage and dissociation of hydrogen, and palladium (Pd) is a conventional hydrogen storage metal. Dissociation is assumed to take place on the palladium particles and atomic hydrogen spills over to the HNTs for the Pd-HNTs sample. The atomic and molecular hydrogen could be adsorbed within the HNTs matrix by physical adsorption. Owing to their high adsorption capacity and high adsorption/desorption rates, the carbon materials could be predominantly microporous and have to provide an adequate surface chemistry and good mass transfer properties. Incorporation of carbon into another matrix material to form a new binary support material for hydrogen spillover can be more effective for hydrogen storage^19^,^20^. The two hybrid nanotubes, Pd-3C-HNTs and 3C-Pd-HNTs display remarkable hydrogen storage capacities as the hydrogen adsorption capacity of Pd-3C-HNTs is 0.58 wt% at room temperature and 2.65 MPa, and that of 3C-Pd-HNTs is 0.63 wt% derived from the isotherms. The atomic and molecular hydrogen could be adsorbed within the HNTs and carbon layer supports by physical adsorption, and the transmission of atomic and molecular hydrogen is unidirectional first from Pd nanoparticles to the carbon layer and then to the HNTs substrates for Pd-3C-HNTs sample. It is worth noting that 3C-Pd-HNTs exhibits the steepest increase in the hydrogen uptake when compared with the uptake profiles of the other samples. The remarkable enhancement was ascribed to the superior surface area of the 3C-Pd-HNTs sample because carbon coating has a positive correlation with the specific surface area within limits (S_BET_ = 49.07 m^2^/g for Pd-HNTs, while S_BET_ = 64.56 m^2^/g for 3C-Pd-HNTs) and the special location of Pd nanoparticles for bidirectional transmission of hydrogen. For the 3C-Pd-HNTs sample, dissociation is also assumed to take place on the palladium particles and atomic hydrogen spills over to the HNTs and carbon layer supports at the same time, and the transmission of atomic and molecular hydrogen is bidirectional as the Pd nanoparticles are between the carbon layer and HNTs. The bidrectional spillover can create more intimate contact between the metal particle and the carbon-HNTs double supporters, which also contributes to primary spillover enhancement. As the spillover of hydrogen from Pd nanoparticles is multidirectional, it could reduce the spillover to the environment when the Pd nanoparticles are covered with excess carbon and confined between carbon layer and HNTs (inset of Fig. 6). Highly dispersed Pd nanoparticles confined in the interspaces between the carbon layer and HNTs plays an important role in increasing the hydrogen storage. For Pd-HNTs, the spillover hydrogen was only stored within HNTs. After carbon coating, the spillover hydrogen was stored in micro-pores formed by carbon coating, leading to the higher hydrogen adsorption capacity. It implied that the coated carbon provides an efficient storage space for spillover hydrogen, highly dispersed Pd nanoparticles confined in the interspaces between the carbon layer and HNTs play an important role in increasing the hydrogen storage. A certain amount of carbon enter into the interspace between spherical Pd nanoparticle and HNTs, and act as a physical bridge which can reduce the physical/energy barriers for surface diffusion of hydrogen atoms form the noble metal nanoparticles site to the receptor, while confining the metal particles in the system to creat physical bridges is helpful for increasing the contacts and, hence facilitating the primary and secondary spillover^19^,^20^. The results suggest that the characteristics of supporters, and the combination of Pd and supports are the predominant factors in determining the overall hydrogen adsorption capacity. The carbon and HNTs receptors are important factors influencing the hydrogen storage via spillover hydrogen. For this spillover mechanism of Pd-carbon-HNTs ternary system in this study, the bidirectional transmission of atomic and molecular hydrogen is concluded to be more effective than the unidirectional transmission.

In summary, hybrid nanotubes with highly ordered structures and large surface areas were successfully synthesized via the hydrothermal method from natural halloysite nanotubes (HNTs). The study demonstrates that the herein studied HNTs and hybrid nanotubes samples are promising mediums for physisorption-based hydrogen storage at room temperature. HNTs showed a high hydrogen adsorption capacity of 0.35 wt%, measured at room temperature and 2.65 MPa, and that of 3C-HNTs is as high as 0.48 wt%. These are among the highest reported values of nanotubes in the literatures obtained under the same conditions. The results have established the importance of building of carbon layer for enhancing hydrogen storage capacity of HNTs for the hybrid nanotubes. Large surface area, plenty of mesopores, and appropriate small average pore diameter are in favor of hydrogen adsorption for *x*C-HNTs samples. By introducing a small amount of Pd into the hybrid nanotubes, improved hydrogen adsorption capacities were obtained through the spillover mechanism. Two different formations of spillover of hydrogen were also investigated for contrast. The hydrogen adsorption capacities are 0.58 wt% and 0.63 wt% under the same condition (room temperature and 2.65 MPa) for Pd-3C-HNTs and 3C-Pd-HNTs, respectively. The bidirectional transmission of atomic and molecular hydrogen is concluded to be superior to the unidirectional transmission. This spillover mechanism of orientation could offer a benign approach for the synthesis of noble metal functionalized ternary composite materials which could be potentially used as hydrogen storage medium at room temperature.

## Methods

### Preparation

Natural halloysite (HNTs) was collected from Hunan, China. All of the chemicals were of analytical grade and were used without further purification. The natural halloysite nanotubes were first emulsified with distilled water and then collected via filtration, washed with deionized water, and finally dried at 60 °C for 8 h. (1) Hybrid nanotubes of carbon and natural halloysite were prepared according to the procedure: 1.2 g of HNTs and the appropriate amount of sucrose were dissolved in the mixed solution of deionized water and ethanol. The suspending solution was transferred to a Teflon-lined steel autoclave and statically heated at 170 °C for 24 h after ultrasonic dispersion for 30 min and magnetic stirring for 2 h. The resultant brown-black powder was filtered, washed with deionized water and ethanol for three times, and dried at 80 °C overnight. The obtained powders during these procedures are the precursors of the target products (3C-HNTs and 5C-HNTs), named as P-3C-HNTs and P-5C-HNTs, respectively. The powder was transferred into a vertical quartz tube, and then calcined at 600 °C for 3 h with the heating rate of 5 °C/min under nitrogen atmosphere. After calcination, the color of samples changed from brown-black to totally black. After cooling down to room temperature under nitrogen atmosphere, the final products of hybrid nanotubes were stored and denoted as *x*C-HNTs (*x* = 1, 3 and 5 when the pre-set C:HNTs mass ratios are 1:1, 3:1, 5:1, respectively). (2) Palladium-modified HNTs sample (Pd-HNTs) was synthesized as follows: first, 83.31 mg of palladium chloride (PdCl_2_) and 0.15 g of polyvinyl pyrrolidone were dissolved in 50 mL of methanol and refluxed for 2 h at 68 °C to form a dark brown solution (PVP-PdCl_2_ solution). Then, 1 g of the HNTs was kept in 50 mL of the PVP-PdCl_2_ solution for 12 h and then rinsed thoroughly with deionized water. After dried at 80 °C, the color of the sample changed from white to brown. Finally, the impregnated sample was reduced by 100 mL of 0.0375 M alkaline solution of hydrazine hydrate (N_2_H_4_·H_2_O) and completely washed with deionized water, the color of the sample changed from brown to grey-black suggesting the formation of metallic palladium in the sample. (3) Palladium modified hybrid nanotubes with the C:HNTs mass ratio of 3:1 (Pd-3C-HNTs) was synthesized with the same procedures as Pd-HNTs, except that the source of HNTs changed to 3C-HNTs. (4) Hybrid nanotubes with palladium nanoparticles inside two phases with the C:HNTs mass ratio of 3:1 (3C-Pd-HNTs) was synthesized with the same procedures as 3C-HNTs, except that the powder of HNTs changed to Pd-HNTs.

### Characterization

X-ray diffraction (XRD) patterns were recorded on Rigaku D/max 2550 with Cu Kα radiation (λ = 0.15406 nm) over a scanning range of 2*θ* = 5–80° with a step width of 0.02°, and at a voltage of 40 kV and a current of 200 mA. Nitrogen adsorption-desorption isotherms were obtained at −196 °C, using a Micromeritics ASAP 2020 equipment. All the samples were vacuum—dried at 150 °C for 8 h prior to the measurements. The specific surface area (S_BET_) was determined from the isotherms by the Brunauer-Emmet-Teller (BET), Langmuir, t-plot methods, and the total pore volume was obtained from the maximum amount of nitrogen gas adsorbed at a partial pressure, *p*/*p*_0_, above 0.99. The pore size distribution was calculated by the Barrett-Joyner-Halenda (BJH) method, using the nitrogen adsorption branch of the isotherm. Transmission electron microscopy (TEM) images were recorded on a JEOL JEM-2100F electron microscope, fitted with an energy dispersive X-ray (EDX) analyzer, at an accelerating voltage of 200 kV. For sample measurements, the powder samples were dispersed in ethanol, as assisted by ultrasonic dispersion for several minutes. The resulting suspension was dripped onto a carbon-coated copper grid and allowed to naturally dry in air. Fourier transform infrared (FTIR) absorption spectra of the samples were measured by a Nicolet NEX-US 670 IR spectroscopy, analytical grade KBr was used as dispersant and range of the spectrum was settled from 400 to 4000 cm^−1^. X-ray photoelectron spectroscopy (XPS) measurements were performed on a Thermo Fisher Scientific K-Alpha 1603 spectrophotometer with Al Kα radiation (1486.6 eV).

### Hydrogen uptake measurements

The hydrogen adsorption isotherms were measured using a static volumetric technique with a specially designed Sieverts apparatus at atmospheric temperature, 25 °C^40^. The testing pressures were set to a range of 0.4 to 2.8 MPa. Prior to the measurements, the apparatus was examined for leakage and calibrated at 25 °C. The dried testing cell with sample loaded was degassed at 240 °C and at a pressure of no more than 4.0 Pa. Then hydrogen activation was conducted at a pressure of 1.2 MPa and a temperature of about 140 °C to remove other surface-adsorbed gases. Prior to measurements, the sample was further degassed at 240 °C (pressure < 4.0 Pa). After the sample cell cooled down to 25 °C, pure hydrogen was injected in the test system at different pressures to measure the hydrogen adsorption capacity.

## Additional Information

**How to cite this article**: Jin, J. *et al.* Carbon hybridized halloysite nanotubes for high-performance hydrogen storage capacities. *Sci. Rep.* **5**, 12429; doi: 10.1038/srep12429 (2015).

## Supplementary Material

Supplementary Information

## Figures and Tables

**Table 1:** Porous parameters of the samples.

Samples	BET surface area (m^2^/g)	Langmuir surface area (m^2^/g)	t-plot external surface area (m^2^/g)	Pore volume (cm^3^/g)	Average pore diameter (nm)
HNTs	64.52	62.51	64.52	0.200	12.45
1C-HNTs	60.28	57.15	60.28	0.219	14.56
3C-HNTs	47.14	43.13	47.14	0.146	12.35
5C-HNTs	30.35	28.97	30.35	0.128	16.93
Pd-HNTs	49.07	44.32	49.07	0.182	14.87
Pd-3C-HNTs	32.59	30.08	32.59	0.137	16.78
3C-Pd-HNTs	64.56	59.35	64.56	0.293	18.13
