# Supplementary material for: Carbon hybridized halloysite nanotubes for high-performance hydrogen storage capacities
Source: Sci Rep. 2015 Jul 23;5:12429. doi: 10.1038/srep12429 (PMC5378895; doi:10.1038/srep12429)
Supplement: Supplementary Information [file srep12429-s1.pdf]

Supplementary Information

Carbon hybridized halloysite nanotubes for high-performance hydrogen storage capacities

Jiao Jin[+], Liangjie Fu[+], Huaming Yang[*], Jing Ouyang[*]

Centre for Mineral Materials, School of Minerals Processing and Bioengineering, Central South University, Changsha 410083, China

[*] *Corresponding author, E-mail:hmyang@csu.edu.cn, jingouyang@csu.edu.cn; Tel: +86-731-88830549, Fax: +86-731-88710804*

[+] *These authors contributed equally to this work*

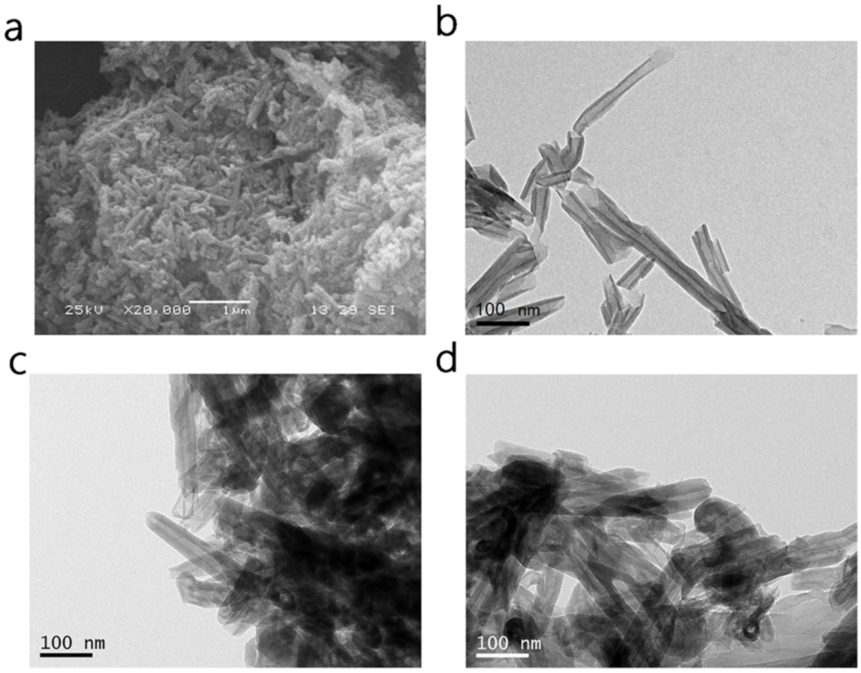

Figure S1 Morphologies of the samples. (a) SEM images of HNTs; TEM images of (b) HNTs, (c) 1C-HNTs and (d) 3C-HNTs.

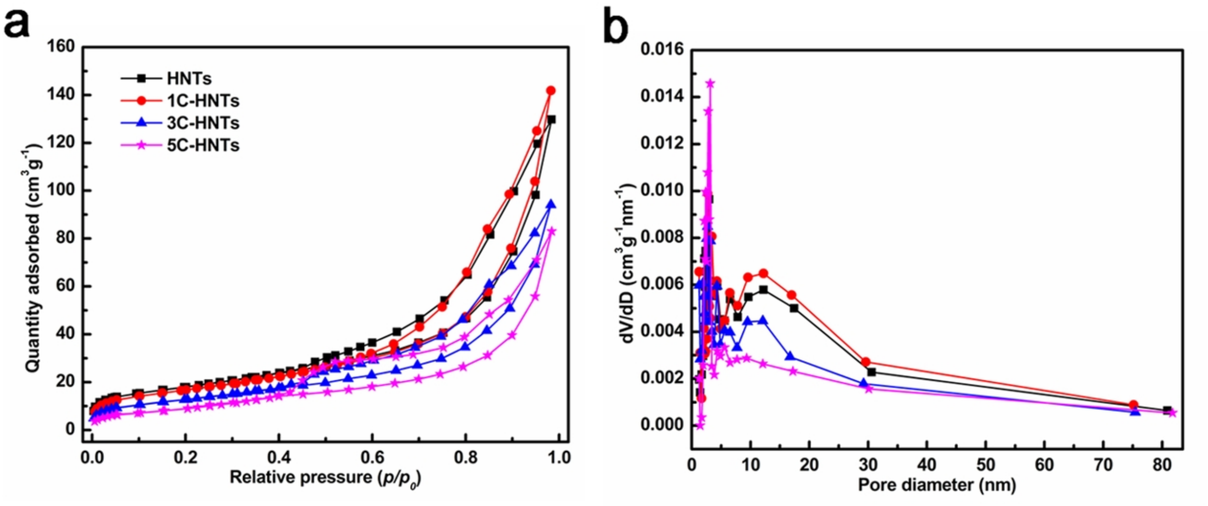

Figure S2 Porous textures of the samples. (a) Nitrogen adsorption-desorption isotherms and (b) pore size distributions of HNTs, 1C-HNTs, 3C-HNTs and 5C-HNTs.

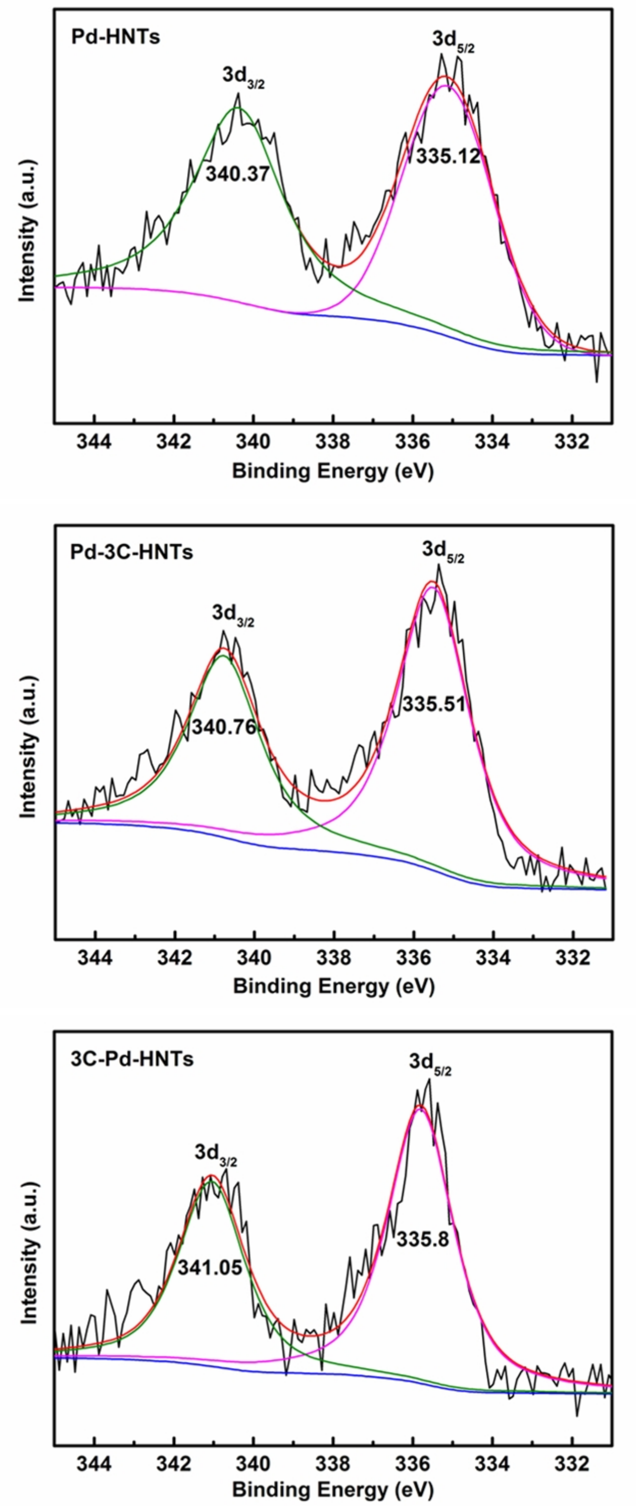

Figure S3 Pd 3d XPS spectra analysis of the samples.

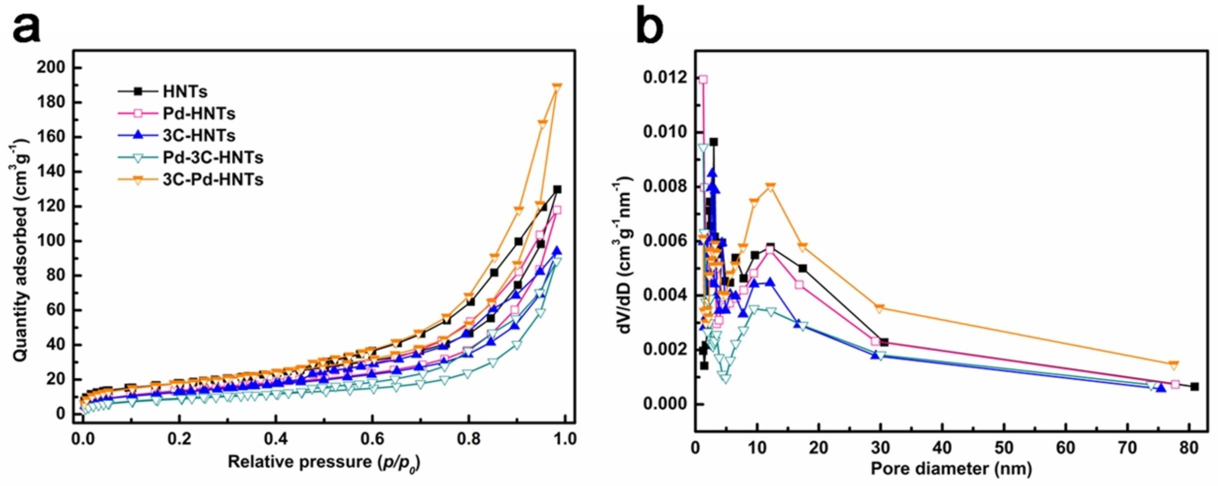

Figure S4 Porous textures of the samples. (a) Nitrogen adsorption-desorption isotherms and (b) pore size distributions of HNTs, Pd-HNTs, 3C-HNTs, Pd-3C-HNTs, and 3C-Pd-HNTs.
